# Role of Surgical and Medical Management of Chronic Thromboembolic Pulmonary Hypertension: A Systematic Review

**DOI:** 10.7759/cureus.53336

**Published:** 2024-01-31

**Authors:** Alexandra V Crowley, Megan Banfield, Aditi Gupta, Rhea Raj, Vasavi R Gorantla

**Affiliations:** 1 Medicine, St. George's University School of Medicine, True Blue, St. George's, GRD; 2 Biomedical Sciences, West Virginia School of Osteopathic Medicine, Lewisburg, USA

**Keywords:** pulmonary hypertension, anticoagulants, balloon pulmonary angioplasty (bpa), pulmonary endarectomy (pea), thrombus, chronic thromboembolic pulmonary hypertension (cteph)

## Abstract

Chronic thromboembolic pulmonary hypertension (CTEPH) is underdiagnosed and has recently surfaced as one of the leading triggers of severe pulmonary hypertension. This disease process is described by structural changes of pulmonary arteries such as fibrous stenosis, complete obliteration, or the presence of a resistant intraluminal thrombus, resulting in increased pulmonary resistance and eventually progressing to right-sided heart failure. Hence, this study aims to describe the current treatments for CTEPH and their efficacy in hemodynamic improvement and prevention of recurring thromboembolic episodes in patients. This systematic review promptly follows the Preferred Reporting Items for Systematic Reviews and Meta-analyses (PRISMA) guidelines. On February 13, 2022, our team searched through the following databases: PubMed, ProQuest, and ScienceDirect. The following keywords were used across all databases: CTEPH AND Pulmonary Endarterectomy (PEA), CTEPH AND Balloon Pulmonary Angioplasty (BPA), and CTEPH AND Medical Therapy OR Anticoagulation therapy. Twenty-nine thousand eighty-nine articles on current management techniques (PEA, Balloon angioplasty, anticoagulants) were selected, analyzed, and reviewed with each other. We found 19 articles concerning PEA, 15 concerning BPA, and six regarding anticoagulants. Most papers showed high success rates and promising evidence of PEA and anticoagulants as a post-operative regimen. BPA was the least preferred but is still reputable in patients unfit for invasive techniques. CTEPH is a condition presenting with either fibrous stenosis, complete obliteration of the artery, or a clogged thrombus. Recent studies have shown three techniques that physicians have used to treat CTEPH: balloon-pulmonary angioplasty, PEA, and medical management with anticoagulants. PEA followed by anticoagulants is preferred to balloon pulmonary angioplasties. CTEPH is an ongoing topic in research; as it continues to be researched, we hope to see more management techniques available.

## Introduction and background

Chronic Thromboembolic Pulmonary Hypertension (CTEPH) is one of the notorious causes of severe pulmonary hypertension (PH) [1]. CTEPH is often described as an area of coagulation [1,2] within the vessels with fibrous stenosis or complete blockage of pulmonary arteries [1]. CTEPH can affect any group of people with a history of pulmonary embolism diagnosis, particularly older adults [1]. Risk factors for CTEPH include age >70 years and systolic pulmonary artery pressure >50 mmHg at initial presentation [1]. One of the latest studies suggested that the prevalence of CTEPH varies from 1% to 3.8% of patients two years after an occurrence of acute pulmonary embolism [2]. The underlying pathogenesis of CTEPH is the disruption of efficient perfusion of cells and tissues of the body with adequate oxygen. If it is not treated, patients can die within a few years by progressive worsening clinical presentations such as cyanosis and severe dyspnea. Various treatments are available for CTEPH, though some may provide better results than others. 

The removal of blood clots via pulmonary endarterectomy (PEA) is one of the available treatment options for patients with CTEPH. It has been demonstrated to significantly improve hemodynamics and prognosis with a three-year survival rate of 89% compared to the 70% survival rate of those who did not undergo PEA [3]. However, with approximately 40% of cases being considered inoperable due to the location and accessibility of the occlusion, age, and comorbidities that may increase the risk of this complex surgery, PEA may not be a viable option for every patient [4,5]. Despite its notable efficacy, PEA has also been associated with potentially detrimental postoperative complications such as persistent PH in 5% to 35% of patients [6].

It is thus imperative that alternate treatments are considered, such as Balloon Pulmonary Angioplasty (BPA) - a reputable treatment in patients with Congenital Pulmonary Stenosis that is recently evolving as a potentially successful therapy for those with CTEPH who are unfit for the more invasive preference of PEA [7,8]. BPA is a catheter-based treatment, which has also been disclosed to be an adequate alternate decision for this group of patients [9]. It is a percutaneous approach intended to widen or enlarge stenotic openings in obstructed pulmonary arteries with a balloon catheter technique accompanied by fluoroscopy [7]. BPA with small balloons over guide wires principally disintegrates mesh or entanglements within the lumen of the blood vessel without disrupting vessel layers [10]. Generally, repeat sessions of BPA are required for the best clinical outcome [10].

With continued studies and clinical success, BPA can be a promising alternative to other procedures. Yet, it has been over 20 years since the initial reports of BPA, and even with good research available, it is still not entirely accepted as a therapeutic route for all inoperable patients with CTEPH [11]. More research is desired in this field.

Another treatment is anticoagulation therapy. As the term “anticoagulant” suggests, it is a substance that prevents the formation of a thrombus or blood clot, which is present in patients with CTEPH [12]. Such pharmacologic therapy improves hemodynamics and delays clinical worsening in inoperable patients and persistent PH in those receiving PEA [13]. Standard medical treatment includes vitamin K antagonists (VKA) [14]. However, there are limitations, such as constant monitoring of bleeding events, changes in diet, and certain medications [1]. A study done by Martin and Cuttica highlighted research that compared the anticoagulation abilities of warfarin and direct-acting oral anticoagulants (DOACs) and revealed a higher incidence of venous thromboembolism recurrence with DOACs (4.62%/person-year) than compared with VKA (0.76%/person-year) [14]. Additionally, there was no difference in survival or significant bleeding events using either DOACs or VKA [14]. Their study also suggested implementing VKA for six months following surgical pulmonary thromboendarterectomy as the recurrence of venous thromboembolism occurred after five to six months post-operation [14].

Riociguat is currently approved as the only therapy used in patients with inoperable or recurrent CTEPH [15]. It is a soluble guanylate cyclase stimulator, thus actively converting GTP to cyclic guanosine monophosphate (cGMP), which is an essential second messenger in the nitric oxide pathway - the mechanism of Riociguat [13]. Nitric oxide leads to arterial vasodilation, decreases pulmonary vascular resistance (PVR), and improves the 6-minute walk distance [14].

The following systematic review seeks to highlight three alternative treatments for CTEPH that have had promising results in patients’ hemodynamics, long-term survival, and quality of life (QOL): PEA, BPA, and medical management with anticoagulation therapy.

## Review

Methods

This systematic review promptly follows the Preferred Reporting Items for Systematic Reviews and Meta-analyses (PRISMA) guidelines [16]. A search for the literature was performed on February 13, 2022, through the following databases: PubMed, ProQuest, and ScienceDirect. The following keywords were used across all databases: CTEPH AND Pulmonary Endarterectomy, CTEPH AND Balloon Pulmonary Angioplasty, and CTEPH AND Medical Therapy OR Anticoagulation therapy. Twenty-nine thousand eighty-nine publications were found and screened based on the inclusion and exclusion criteria described below. The remaining 7,024 publications were manually screened, and only relevant research regarding the research question was considered. A total of 40 articles [1] were kept for our systematic review.

Inclusion Criteria

The studies selected for this systematic review were selected and included based on the following factors: articles were clinical trials and meta-analyses written in English, peer-reviewed, full-text, published in the previous 20 years (2002-2022), and applied to our topic. COVID-19 studies were also included with the same filters described to show current relevance to the pandemic.

Exclusion Criteria

**Figure 1:**
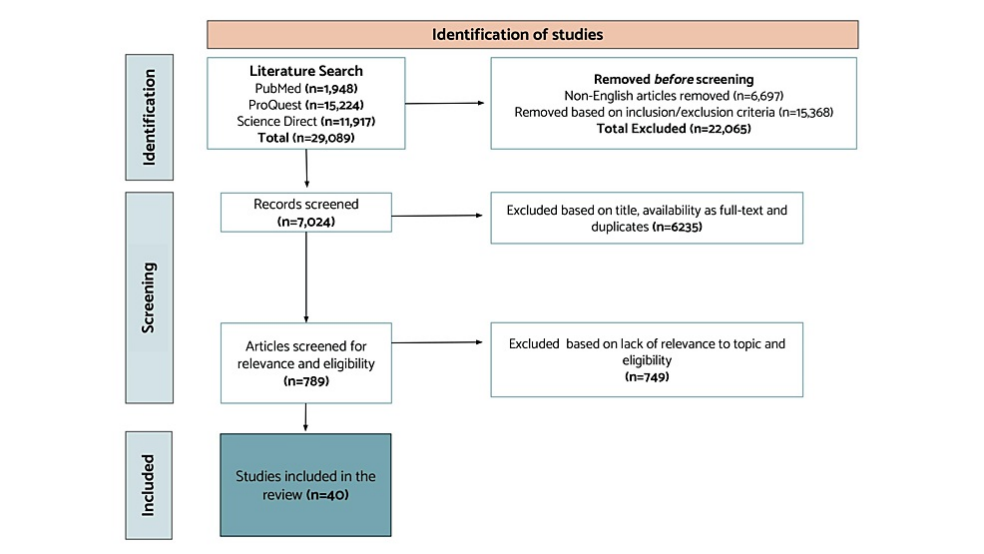
PRISMA flowchart illustrating the screening process of studies related to surgical versus medical management of CTEPH

We excluded case reports, case studies, narrative reviews, duplicates of included articles, studies not written in English, or those not applicable to our topic from the literature review (Figure 1).

Results

Conclusively, 40 articles were used for our final review. Our final evaluation included 19 for PEA, 15 for balloon angioplasty, and six for medical therapy (anticoagulation). All the articles included generally focused on the three standard therapies in treating CTEPH (Table 1).

**Table 1:** Studies on the role of surgical and medical management of chronic thromboembolic pulmonary hypertension (CTEPH) Table showing a summary of articles on the role of surgical (BPA and PEA) and medical (anti-coagulation therapy) management of CTEPH. Studies are organized in order of reference throughout the paper. Some studies focus on the immediate hemodynamic improvements of patients, while others focus on long-term outcomes, survival rates and possible risks each treatment plan may pose. BPA: Balloon Pulmonary Angioplasty, PEA: Pulmonary Endarterectomy, NSE PTA: non-slip element percutaneous transluminal angioplasty,  PVR: pulmonary vascular resistance, mPAP: mean pulmonary arterial pressure, VTE: venous thromboembolism, QOL: quality of life, 6MWD: 6-minute walking distance, COVID-19: Coronavirus Disease of 2019, WHO FC: World Health Organization and Functional Class

	Author	Country	Design & Study Population	Aim	Findings	Conclusion
1	Taniguchi et al., 2020	Japan	Retrospective study [n=143]	To examine the efficacy of BPA vs. PEA in relation to long-term survival	The 1, 3, and 5-year survival rates for the BPA vs. PEA group were as follows: 97.6%, 93.0%, 90.1% vs. 92.9%, 85.1%, 74.5%. There were no mortalities in patients treated via combination therapy.	BPA and PEA are equally efficient treatment choices for CTEPH, but combining both may drastically improve outcomes.
2	Hsieh et al., 2018	Czech Republic	Meta-analysis [n=4868]	To evaluate the rate of postoperative outcomes of PEA, such as persistent pulmonary hypertension	25% of CTEPH patients experienced persistent pulmonary hypertension following PEA	Persistent pulmonary hypertension is a common postoperative outcome in those with CTEPH
3	Freed et al., 2011	United Kingdom	Prospective Study [n=314]	To investigate the effect of persistent pulmonary hypertension post-PEA	Patients with persistent pulmonary hypertension (PH) post-PEA experienced a reduction in exercise capacity and improvement in symptoms but minimal difference in survival at five years compared to those without PH (89.9% vs. 90.3%, respectively)	Persistent pulmonary hypertension leads to impaired hemodynamic improvement post-PEA but does not have an effect on medium-term survival
4	Ogawa et al., 2017	Japan	Retrospective Study [n=308]	To confirm the hemodynamic improvements after balloon pulmonary angioplasty (BPA) using a retrospective technique	Overall, survival was 96.8% at one and two years and 94.5% at three years, respectively, after the initial BPA procedure in all 308 patients.	The multicenter registry suggested amended hemodynamic results after BPA procedures. Complication rates were high, but overall survival was as good as that of patients who received pulmonary endarterectomy.
5	Kawakami et al., 2020	Japan	Randomized Controlled Trial [n=60]	Evaluate the effectiveness and safety of BPA and another treatment known as Riociguat over a relatively more extended period (12 months)	For the primary analysis, the least square means difference and 95% CI for change of pulmonary arterial pressure between groups at 12 months.	BPA is an additional option of treatment among other treatments that have worked in other patients, such as Riociguat
6	Mizoguchi et al., 2012	Japan	Clinical Trial [n=68]	To distinguish and resolve two major problems that need to be resolved to better the clinical efficacy of BPA.	Forty-one patients (60%) developed reperfusion pulmonary injury after BPA, but mechanical ventilation was required in only four patients.	Their refined BPA procedure enhances the clinical status and hemodynamics of inoperable patients with CTEPH and a lower mortality rate
7	Van Thor et al., 2019	The Netherlands	Retrospective study [n=36]	To describe the long-term clinical outcome of Riociguat therapy in patients with inoperable and/or residual CTEPH	Twenty-four patients experienced at least one adverse event, such as headaches, diarrhea, upper respiratory tract symptoms, anemia, and dyspepsia. Seven patients experienced overall clinical worsening, and two died after clinical worsening. Thus, Riociguat helped the majority of inoperable patients. WHO FC improved, as well as the 6-minute walking distance test.	Riociguat is an effective long-term therapeutic anticoagulant with rational survival and improved clinical parameters.
8	Humbert, 2022	USA	Prospective study [n=968]	To monitor the long-term outcomes of Riociguat in clinical practice and compare the effects of NOACS with VKAs.	General adverse events were reported in 438 patients under VKA and 135 patients in the NOAC group.	There were similar incidences of bleeding events between patients receiving VKA and NOACs, with NOACs having a slightly higher rate.
9	Ghofrani et al., 2021	28 countries worldwide (EXPERT study)	Prospective uncontrolled study [n=956]	To monitor the long-term outcomes of Riociguat in a clinical setting.	In Riociguat, newly treated patients had a shorter CTEPH-symptom exacerbated episode than those with improved clinical parameters.	No new safety concerns were identified, and the results were consistent with current research on Riociguat.
10	Kramm et al., 2005	Germany	Double-blinded randomized trial [n=22]	To assess the efficacy of inhaled iloprost in improving hemodynamics in those with persistent pulmonary hypertension post-PEA	Patients treated with inhaled iloprost post-PEA experienced increased cardiac index (difference of +0.6 ± 0.1 L min^-1 ^m^-2^ vs placebo: -0.04 ± 0.1 L min^-1 ^m^-2^) and decreased pulmonary vascular resistance (difference of -183 ± 88 dyn s cm^-5^ vs placebo: -16 ± 38 dyn s cm^-5^)	Iloprost administration post-PEA can successfully improve persistent pulmonary hypertension following PEA
11	Mayer et al., 2011	Germany	Prospective study [n=679]	To assess the surgical management and outcomes of CTEPH patients enrolled in an international registry	Patients assessed one-year post-PEA showed decreased pulmonary vascular resistance (698 to 235 dyn s cm^-5^) and an increased 6-minute walking distance (6MWD; 362 to 459 m). The in-hospital mortality rate was 4.7%.	PEA provides substantial improvement in hemodynamic profile and is correlated with a low in-hospital mortality rate
12	Liu et al., 2021	Taiwan	Retrospective study [n=27]	To assess patient outcomes following PEA	Post-PEA, the 6MWD increased by 60.5%, and survival rates related to the disease were 96.3%	Patient hemodynamic symptoms were ameliorated following PEA, improving them to New York Association functional class I and II and providing them with a more favorable prognosis
13	Casaclang-Verzosa et al., 2006	United States	Clinical Trial [n=32]	To assess the effects of PEA on various echocardiographic parameters in CTEPH patients	Right-sided echocardiographic parameters showed significant improvement three months after PEA: Right-ventricular end-diastolic area (mean difference of 5.8 ± 10.4 cm^2^) and end-systolic area (mean difference of 6.3 ± 10.01 cm^2^), right ventricular systolic pressure (mean difference of (40.0 ± 24.8 mmHg), and tricuspid regurgitation (mean difference in grade of 1.5 ± 1.0)	PEA is associated with marked and sustained improvement in right-sided echocardiographic parameters
14	Marston et al., 2014	United States	Retrospective study [n=48]	To evaluate the beneficial effects of PEA on left atrial filling	Following PEA, the left atrial volume index increased by 18%, along with positive changes in pulmonary vascular resistance and cardiac index	Successful PEA can lead to improvements in left atrial filling
15	Kato et al., 2016	Japan	Retrospective study [n=117]	To investigate the effects the coagulation-fibrinolysis system could have on the outcomes of CTEPH patients following PEA and whether PEA could positively affect the coagulation-fibrinolysis system	Patients with high fibrinogen levels and low plasminogen levels pre-PEA had a significantly lower survival rate of 84% compared to 100% of the other patients. Following PEA, thrombomodulin and plasminogen values were notably increased.	Elevated fibrinogen and decreased plasminogen levels are associated with a dramatic reduction in survival post-PEA. PEA provides beneficial effects for the coagulation-fibrinolysis system.
16	Kallonen et al., 2020	Sweden	Observational cohort study [n=100]	To examine survival rates of CTEPH following PEA	Patients who successfully underwent the operation and postoperative phase had a 59% survival rate at 15 years compared to the survival rate of the general population of 71%	Although patients had a reduced survival rate following PEA, the difference was relatively small, and the prognosis was improved overall.
17	Kim et al., 2016	United States	Randomized controlled trial [n=261]	To evaluate the effects of Riociguat on the hemodynamics of patients with inoperable CTEPH or persistent CTEPH post-PEA	Riociguat effectively reduced pulmonary vascular resistance (-285 dyn s cm^-5 ^and -131dyn s cm^-5^) and mean pulmonary artery pressure (-4.7 mmHg and -4.8 mmHg) and increased the cardiac index (+0.6 min^-1 ^m^-2 ^and +0.2 min^-1 ^m^-2^) in those with inoperable or persistent CTEPH post-PEA	Riociguat is effective in improving the hemodynamics of patients with inoperable or persistent CTEPH
18	Jujo et al., 2016	Japan	Observational retrospective study [n=23]	To assess whether pulmonary arteriopathy is linked to hypoxemia and persistent pulmonary hypertension, two common postoperative complications following PEA	Pulmonary arteriopathy may lead to increased alveolar-arterial oxygen difference and impaired diffusion capacity post-PEA, according to the observed negative correlation between severity of pulmonary arteriopathy and PaO_2_ (r=-0.73 postoperatively and r=-0.66 at follow-up vs. r=-0.373 preoperatively)	Pulmonary arteriopathy was associated with hypoxemia following PEA, but the exact mechanisms remain unclear
19	Reesink et al., 2007	Netherlands	Observational Retrospective Study [n=50]	To evaluate the association between the 6-minute walk distance (6MWD) and the hemodynamic profile of CTEPH patients and the subsequent effect of PEA on this parameter	Patients experienced an increase in the 6MWD (417 ± 19 m to 517 ± 16m), correlating with their improvement in hemodynamics after one year following PEA (BNP: r=0.57, mPAP: r=0.52, CO: r=0.70 and total pulmonary resistance: r=0.70)	The 6MWD is an effective parameter of the hemodynamic profile and symptom improvements in CTEPH patients
20	Ruigrok et al., 2020	Netherlands	Observational retrospective study [n=33]	To investigate the prevalence of thrombotic lesions following PEA and associated hemodynamic implications	27% of patients presented with recurrent lesions within 6 months following PEA with no consequent differences in the hemodynamic profile compared to patients without recurrent lesions.	Recurrent thrombotic lesions are common in CTEPH patients following PEA, but there is no evidence of consequent hemodynamic implications associated with them.
21	Ishida et al., 2012	Japan	Retrospective study [n=77]	To assess the long-term outcomes and the factors affecting those outcomes following successful PEA	Patients with post-PEA mean pulmonary arterial pressure lower than 34 mmHg had significantly better outcomes and freedom rates from disease-specific complications than those with higher values	While PEA can lead to drastic improvements in hemodynamics and prognosis, high pulmonary vascular resistance and mean pulmonary arterial pressure have an increased risk of postoperative complications
22	Thistlethwaite et al., 2006	United States	Retrospective study [n=743]	To examine whether PEA would be beneficial to patients with extreme pulmonary artery systolic pressure (greater than 100 mm Hg)	Patients with pulmonary artery systolic pressures over 100 mmHg experienced a drastic improvement in their hemodynamics compared to those with values under 100 mmHg (mean decrease in PVR: 926.7 ± 511.1 vs 546.4 ± 365.1 dynes. sec cm^-5^)	PEA can have substantial beneficial effects on patients with extreme pulmonary artery systolic pressure, but these patients experienced a higher incidence of major postoperative adverse events
23	Wieteska et al., 2016	Poland	Clinical Trial [n=112]	To investigate the long-term outcomes of CTEPH patients treated via PEA versus medical treatment.	Patients treated with PEA showed a long-term mortality rate of 12.7% compared to medically treated patients (34.8%)	PEA provides a much more favorable prognosis for CTEPH patients than medical treatment alone.
24	Reesink et al., 2010	United States	Randomized controlled trial [n=26]	To assess the effects of bosentan on the hemodynamics and functional capacity in patients in preparation for PEA	When compared to the control group, patients treated with bosentan showed a greater reduction in total pulmonary resistance (mean difference: 299 dynes s cm^-5^), mean pulmonary artery pressure (mean difference: 11 mmHg), and mean right atrial pressure (mean difference: 3.8 mmHg) and showed no evidence of adverse hemodynamic effects post-PEA	Treatment with bosentan pre-PEA may be associated with a more favorable postoperative outcome, serving as a “therapeutic bridge”
25	Castro et al., 2020	Brazil	Retrospective study [n=108]	To assess the possible beneficial effects of medical therapy on hemodynamics prior to PEA	When treated with medical therapy prior to PEA, patients with a more severe hemodynamic profile showed improved survival (89.1% vs 64.1%), but differences in survival compared to the non-medical therapy group were insignificant (87.8% vs 80.3%, respectively).	Medical therapy prior to PEA may optimize the hemodynamic profile in severe cases but provides no significant advantage when compared to the group not receiving therapy.
26	Brenot et al., 2019	France	Clinical Trial [n=154]	To assess the safety and efficacy of balloon pulmonary angioplasty (BPA) in a large cohort of patients with chronic thromboembolic pulmonary hypertension (CTEPH)	There was a significant decrease in pulmonary artery pressure and pulmonary vascular resistance by 26% and 43%, respectively. The main complications included lung injury, which occurred in 9.1% of 1006 sessions.	The study affirms that an upgraded BPA strategy improves the short-term symptoms in inoperable CTEPH patients with a sufficient risk-benefit ratio
27	Tatebe et al., 2016	Japan	Clinical Trial [n=55]	To examine the hypothesis that metabolic and renal dysfunctions are affiliated with CTEPH severity and BPA efficacy	Although PEA is the recommended treatment for CTEPH, 37% of patients are deemed inoperable. BPA has been shown to dramatically improve not only pulmonary hemodynamics but also functional status (numbers not available).	BPA may carry numerous beneficial effects in CTEPH patients, not only in terms of hemodynamics but other systemic functions, with positive correlations among them
28	Andreassen et al., 2013	Norway	Observational Cohort Study [n=16]	To examine the effectiveness of balloon pulmonary angioplasty (BPA) on CTEPH in patients with inoperable disease or persistent pulmonary hypertension after pulmonary endarterectomy	The study confirmed that BPA treatment in selected CTEPH patients may decrease PAP and improve hemodynamic profile, enhance functional capacity, and is confined to highly motivated patients because of experimental nature/significant complications. (numbers not available)	BPA may offer an alternative form of treatment in selected CTEPH patients. Significant periprocedural complications must be documented.
29	Matsuoka et al., 2021	Japan	Clinical Trial [n=99]	To clarify the clinical impact of oxygen parameters on BPA outcomes in 99 patients.	Nearly normal hemodynamics was achieved after BPA, oxygenation only slightly improved, and exertional desaturation remained unchanged. Nearly normal hemodynamics was achieved after BPA (mean pulmonary artery pressure: 37.5 +/- 10.0 to 20.6 +/- 4.9 mmHg, p<0.01).	Hemodynamics normalized for the most part; however, oxygen did not. Exertional desaturation remained unchanged, which may be a residual symptom after BPA. This study suggested that domiciliary oxygen therapy should be continued if necessary for the patient.
30	Chen et al., 2020	Taiwan	Retrospective Study [n=13]	To ultimately evaluate the safety and efficacy of BPA in their center in Taiwan and assess early effects on cardiopulmonary function in these CTEPH patients.	WHO functional class significantly improved in all 13 patients, and both mean pulmonary artery pressure and pulmonary vascular resistance significantly decreased. Both mean pulmonary artery pressure and pulmonary vascular resistance significantly decreased from 44.6 +/- 11.7 mmHg to 32.6 +/- 5.1 mmHg (P=0.005).	BPA improved both clinical symptoms and hemodynamic data in the 13 inoperable CTEPH Taiwanese patients.
31	Minatsuki et al., 2021	Japan	Retrospective Study [n=2512 sessions]	To describe the patient and procedural characteristics of BPA performed in Japan using the mandated nationwide registration. In addition, to establish BPA as a safe treatment for all CTEPH patients in Japan.	BPA sessions increased during the study period with acceptable in-hospital complication rates. In-hospital death was observed in 0.2%, and the total complication rate was 5.3%.	With this information, BPA proves to be a feasible and safe therapeutic method for treating patients with CTEPH.
32	Minatsuki et al., 2019	Japan	Retrospective Study [n=43]	To clarify the effectiveness of BPA treatment in inoperable patients with CTEPH.	In all patients, the mean pulmonary arterial pressure, pulmonary vascular resistance, arterial oxygen saturation, and 6-minute walking distance significantly improved after BPA treatment. The mean pulmonary artery pressure from 43.3 +/- 7.8 mmHg to 23.9 +/- 4.7 mmHg; pulmonary vascular resistance, from 924.1 +/- 462.2 dynes etc.	BPA safely improved these measures and the functional statuses of inoperable CTEPH patients.
33	Minatsuki et al., 2020	Japan	Retrospective, Observational Study [n=45]	This study aimed to calculate QOL in a sample of Japanese patients with CTEPH using the EQ-5D and to investigate whether or not BPA would improve it.	The findings revealed that BPA improved QOL scores measured by the EQ-5D in Japanese patients. QOL FROM 0.741 +/- 0.195 to 0.802 +/- 0.160, p<0.05.	BPA improved QOL in CTEPH patients, mainly by improving the “usual activities.”
34	Takigami et al., 2022	Japan	Clinical Trial [n=108]	To evaluate the therapeutic effects and safety of the “non-slip element percutaneous transluminal angioplasty (NSE PTA)” scoring balloons in BPA	No significant differences in the pressure ratios of the two groups.	NSE PTA scoring balloon proved to be safe. However, there was no significant pressure gradient improvement with NSE PTA scoring balloon compared to conventional BPA practices.
35	Fukui et al., 2016	Japan	Clinical Trial [n=41]	To establish the safety and efficacy of cardiac rehabilitation initiated directly following BPA in patients with inoperable CTEPH who presented with continuing exercise intolerance and symptoms on effort even after a course of BPA	No significant between-group differences were found for any baseline characteristics. At week 12, peak oxygen uptake percent predicted peak VO2 (70.7 +/- 9.4% to 78.2 +/- 12.8%, p<0.01).	The grouping of BPA and subsequent CR is a new treatment strategy for inoperable CTEPH to improve exercise capacity to near-normal levels and heart failure symptoms with a good safety outlook.
36	Miura et al., 2021	United States	Prospective Study [n=22]	To investigate whether objective exercise tolerance, evaluated using cardiopulmonary exercise testing and the quantitative QOL score, can be improved after extensive BPA in CTEPH patients	The most striking results revealed that extensive BPA in inoperable CTEPH patients after partial hemodynamic improvement is associated with improved objective exercise tolerance and improved QOL. The peak VO2 showed a significant improvement at entry, finish, and follow-up (17.3+/- 5.5, 18.4 +/- 5.9, and 18.9 +/- 5.3 mL/kg/min, respectively; p <0.001).	Extensive BPA resulted in positive results in exercise tolerance and physical QOL scores. Patients with partially improved hemodynamics benefited as well.
37	Fujii et al., 2021	United States	Pilot Cohort Study [n=45]	To examine whether complete revascularization by additional BPA on residual lesions, even after pulmonary hypertension relief, could resolve hypoxia or the requirement for pulmonary vasodilators	Mean pulmonary arterial pressure and peripheral vascular resistance after the final BPA were further reduced to 18.5 +/- 3.6 mmHg and 171 +/- 65 dyn-s/cm (P<0.001) respectively. These improvements were observed even with the removal of pulmonary vasodilators.	Complete revascularization with BPA beyond pulmonary hypertension relief may improve symptoms, hemodynamics, oxygenation, and exercise capacity
38	Zhu et al., 2021	China	Retrospective study [n=333]	To compare the efficacy and safety of anticoagulant therapy between patients with and without a history of venous thromboembolism, investigate risk factors of recurrence and major bleeding events in CTEPH.	21% of patients without a history of VTE did not experience any recurrent episodes during anticoagulation therapy (0/100 per person per year). Those with a prior history of VTE had an increased recurrence risk (2.27/100 per person per year).	There is a significant direct relationship between having a prior VTE history and recurrent VTE in that patients with a prior VTE history had a greater risk of recurrent VTE than patients with no prior VTE history.
39	Bunclark et al., 2019	United Kingdom	Multicenter retrospective analysis [n=1322 preoperative, 1232 postoperative]	To evaluate outcomes and complications in patients with operable CTEPH post-pulmonary endarterectomy procedure and receiving vitamin K antagonists and direct-acting oral anticoagulants.	794 patients were on a VKA regimen, and 206 were on DOACs after their PEA procedure. After the observation period (approximately 612 days), patients had improved hemodynamics and functional status. Both VKAs and DOACs had the same number of bleeding events, and recurrence was higher in DOACs than VKAs.	Outcomes are unaffected by anticoagulant choice post-PEA. Bleeding events and recurrence were seen in both VKA and DOACs sets of patients however, all had an overall improvement in hemodynamics.
40	Lee et. al, 2020	USA	Cross-sectional survey-based analysis	To assess the total incidence of recognized COVID-19 in patients with CTEPH or PAH to identify the impact of the pandemic on disease progression and management.	Cumulative incidence of COVID-19 with CTEPH was reported at 2.9 cases per 1,000 patients, out of which 30% were hospitalized and 12% died. An increase in the use of telehealth was also seen.	The incidence of COVID-19 in patients with CTEPH was small however, its impact on treatment, testing, and clinical operations was notable.

Many studies in this review highlight PEA as the treatment of choice for CTEPH, with great success in hemodynamic normalization. It is associated with increased long-term survival, even in more severe cases [3,5]. A study by Kato et al. was included that suggests that PEA may benefit the fibrinolytic system, including a significant increase in thrombomodulin and plasminogen values, leading to a more favorable prognosis. One study also showed that successful PEA might result in immediate and sustained right-sided echocardiographic improvements, even after one-year post-surgery. Though the advantages of PEA are evident, it includes the risk of persistent PH. In some cases, the patient may be deemed inoperable and may have to opt for less invasive therapies such as BPA, anticoagulation therapy, or combined therapy.

There have been striking results concerning BPA in the CTEPH realm. Both Brenot et al. and Ogawa et al. recognized that the complication rates in BPA are not particularly low. Still, the survival rates were similar to that of PEA, which was a significant finding [8]. In addition, BPA was shown to substantially improve other factors such as systemic dysfunctions, glycemic control, purine metabolism, renal function, etc. BPA has proved to be successful in those patients who are deemed unfit for surgery; however, even with this research available, this procedure still needs to be accepted as one of the top treatments [11].

Many studies have shown the importance of anticoagulant therapy as the primary long-term management of inoperable CTEPH [12-14]. A few studies have also investigated common concerns with using anticoagulants, such as recurrent episodes of venous thromboembolism and major bleeding events. Some anticoagulant drugs include VKAs (warfarin), DOACs, heparin, and riociguat, each with its respective mechanisms.

Discussion

Pulmonary Endarterectomy

PEA, the gold standard CTEPH treatment, involves median sternotomy and cardiopulmonary bypass with intervals of deep hypothermic circulatory arrest. PEA aims to provide enhancement in hemodynamic parameters such as PVR, mean pulmonary artery pressure (mPAP), right heart function, and cardiac output (CO), as well as prognostic improvement. Some studies have reported on the effect of successful PEA on the normalization of patient hemodynamics, with significant emphasis on PVR and mPAP reduction, which have been notably associated with in-hospital mortality probability post-PEA [5,17,18]. According to a meta-analysis by Hsieh et al., on average, mPAP and PVR were reduced by 21 mmHg and 561 dyn.s/cm5, respectively, following PEA [5]. Research demonstrates that these improvements correlate with patients' advancement from New York Heart Association functional class III/IV to class I/II and a 60.5% increase in patients' 6-minute walking distance (6MWD) [19,20].

Postoperative mortality rates have been found to generally increase with PVR - from as low as 4% in patients with a PVR below 900 dyn.s/cm5 to 20% in those with a PVR above 1200 dyn.s/cm5 [21]. Similarly, mPAP values over 30 mm Hg are associated with a five-year survival rate below 30%, and values over 50 mmHg further reduce the prognosis to 10% [22]. In CTEPH, high PAP values typically increase the workload of the right side of the heart, ultimately leading to right heart failure. Still, studies show that PEA can result in immediate and sustained right-sided echocardiographic improvements at one-year follow-up [23]. Although less commonly described, left ventricular diastolic impairment due to underfilling is frequent in CTEPH and significantly enhanced following PEA with an increase of 18% in left atrial volume index among the typical positive changes usually seen in hemodynamic parameters [24]. Interestingly, PEA may also provide benefits to the fibrinolytic system. Kato et al. found that elevated fibrinogen and decreased plasminogen levels before PEA correlated with the dramatic reduction in prognosis post-PEA (84% compared to 100% of the other patients), potentially as a risk factor for postoperative complications [25]. However, after surgery, patients experienced a marked increase in thrombomodulin and plasminogen values [25]. The drastic enhancement in hemodynamics and, thus, prognosis of CTEPH due to PEA is evident, with patients typically experiencing a 15-year survival rate of 59% compared to that of the general population (71%) [26].

However, surgical treatment with PEA potentiates the risk of frequent and often fatal postoperative complications, such as persistent PH, which is found in approximately 5%-35% of patients [27]. Unrelenting PH is generally due to incomplete resolution of thromboembolic or secondary endothelial changes, such as hyperplasia [5]. These vascular alterations may also contribute to an increased alveolar-arterial oxygen diffusion and impaired diffusion capacity post-operatively [28]. However, the extent of potential postoperative events' implications may be determined by the severity of patient hemodynamics. For example, while patients with unrelenting PH may experience a reduction in exercise capacity and symptom improvement, Freed et al. found minimal difference in survival at five years compared to those without PH (89.9% vs. 90.3%, respectively) [6]. Interestingly, though there may be a relative diminution in exercise capacity (reflected by the 6MWD), studies demonstrate that the absolute increase in 6MWD is higher than those without persistent PH [29]. Other studies show that while 27% of patients present with recurrent lesions within six months following surgery, there are little to no consequent differences in hemodynamic profile compared to patients without lesions [30]. When comparing postoperative outcomes of patients with varying mPAP values post-PEA (lower than 34 mmHg vs. above 34 mmHg), studies show there are correlating differences in 10-year freedom rates from disease-specific death and complications (100% vs. 80% and 98% vs. 41%, respectively) [31].

Yet, hemodynamic severity pre-PEA may not necessarily be a contraindication for surgery. Thistlewaithe et al. discovered that although patients with extreme pulmonary artery systolic pressures (over 100 mmHg) exhibited a higher incidence of detrimental complications such as reperfusion edema, they also showed a more significant reduction in PVR and pulmonary artery systolic pressure when compared to those with values below 100 mmHg [32].

**Figure 2:**
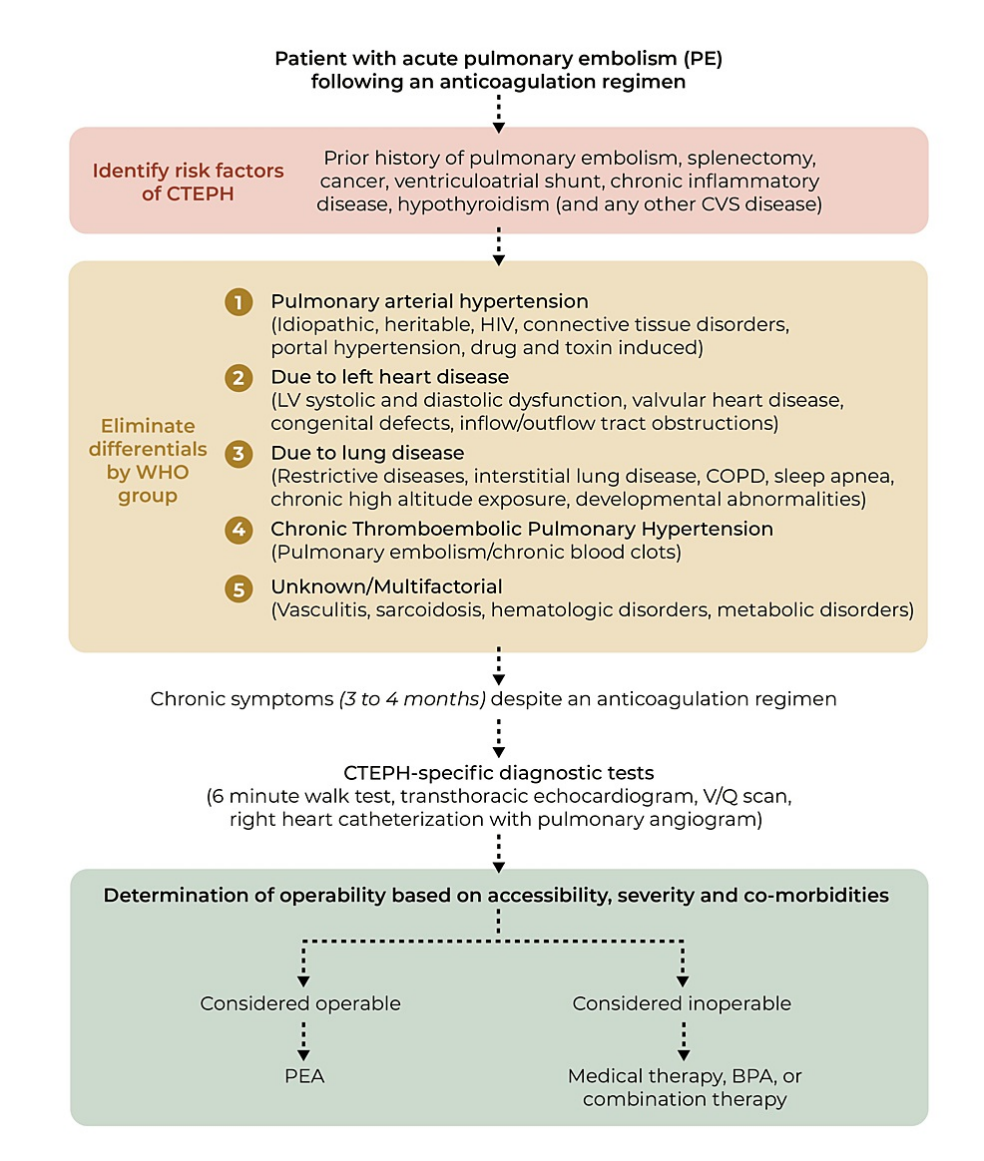
Flowchart illustrating the process of CTEPH diagnosis, evaluation of operability, and the most likely beneficial treatment for a patient. This figure was created by Sue Simon, MS, our Illustrator from St. George's University Abbreviations - CTEPH: Chronic thromboembolic pulmonary hypertension, CVS: Cardiovascular system, V/Q scan: Ventilation and perfusion scan, PEA: Pulmonary endarterectomy, BPA: Balloon angioplasty

Still, 40% of CTEPH cases are rendered inoperable not solely due to age and case severity but, ultimately, the accessibility of the thromboembolic material [4,33]. Indeed, alternative therapeutic strategies must be considered, such as balloon angioplasty (BPA) or medical treatment (Figure 2). Research suggests that a combination of such therapies with surgical intervention may be advantageous, potentially serving as a “therapeutic bridge” via hemodynamic optimization either before surgery or to alleviate persistent PH post-PEA (Figure 2) [3,34,35]. In particular, when treated with a combination of BPA and PEA, patients manifested further hemodynamic enhancement with no mortalities. Likewise, patients with low CO (below 3.75 L/min) treated with pre-PEA medical therapy exhibited a significant prognostic improvement (89.1% one-year survival rate) compared to that of non-treated, low CO patients (64.1%) [35]. Thus, considering combination therapy may be beneficial, especially for higher-risk CTEPH patients.

Balloon Angioplasty

BPA on hemodynamics and oxygen parameters: The current research points to the significant advancements in the BPA technique, which is most evident in Japanese centers. Over recent years, modifications to the method and several limited case series, predominantly from Japan, have delineated noteworthy developments in the safety and value of BPA on clinical outcomes [36]. Brenot et al.’s particular study validated the favorable effects of BPA on hemodynamics with a decline in PVR by more than 50% [36]. Yet, a high complication rate persisted and mainly included non-severe lung injury, resulting in 17.8% of cases [36]. This study confirmed that an altered BPA technique improved short-term symptoms, oxygenation parameters, exercise capacity, and hemodynamics in inoperable CTEPH patients [36].

Ogawa et al. were esteemed as the first multicenter registry to endorse hemodynamic improvement after BPA sessions [8]. Ogawa aimed to verify hemodynamic improvements after BPA using a retrospective technique. The multicenter registry suggested modified hemodynamic results after BP procedures [8]. Similar to Brenot et al., Ogawa et al. also recognized that the complication rate is not particularly low, but the survival rate was similar to that of PEA [8]. Tatebe et al. also elaborated and showed enhanced pulmonary hemodynamics and BPA-boosting long-term prognosis in CTEPH patients [37]. Tatebe further intended to examine how metabolic factors or syndromes play a role in CTEPH severity and BPA efficacy. Their clinical trial revealed that BPA improves pulmonary hemodynamics and the patient's functional status. BPA substantially improved systemic dysfunctions, including glycemic control, purine metabolism, renal function, etc. [37]. In another study that showed promising results, Andreassen et al. validated that BPA treatment in designated CTEPH patients may decrease PAP and improve hemodynamic profiles, tending to stabilize levels of biomarkers to assess proper ventricular function and enhanced functional capacity [38]. Matsuoka et al. aimed to clarify the clinical aspects of oxygen parameters on BPA outcomes and found that they achieved nearly normal hemodynamics after BPA treatment. Oxygenation only slightly improved, and exertional desaturation remained unchanged. Matsuoka et al. suggested that supplementary oxygen therapy should be sustained if crucial for the patient's well-being [39].

BPA safety and efficacy: Chen et al. provided one of the first studies to validate the benefits of BPA in CTEPH patients in Taiwan. They assessed the safety and efficiency of BPA to consider the early effects of cardiopulmonary function in these CTEPH patients. All 13 patients in the study showed significant enhancements and noteworthy decreases in mean arterial pressure (MAP) and PVR [40]. In a different respect, Minatsuki et al. retrospectively wanted to explain the procedural characteristics of BPA and establish it as a safe treatment in Japan specifically. They described BPA as a practicable, safe healing technique for treating patients with CTEPH in Japanese centers [41]. Minatsuki et al. clarified the effectiveness of BPA and found that in all patients, measures such as MAP, PVR, and arterial oxygen saturation safely improved after BPA treatment [42]. A year later, Minatsuki also performed another study on BPA. It improved the QOL scores measured by the EQ-5D in Japanese patients by mainly refining typical activities [43]. Lastly, Kawakami et al. evaluated and focused on the efficacy and safety measures of BPA alongside Riociguat treatment over an extended period. Sequential therapy with Riociguat and BPA for patients diagnosed with inoperable CTEPH has been shown to improve MAP and PVR further emphasizing the benefits of BPA [9]. This study mainly focused on how BPA could benefit the CTEPH patient population by adding an anticoagulant treatment.

Refined BPA procedures: To distinguish between minor issues that need to be resolved to improve the clinical efficacy of BPA, a refined BPA procedure could be considered a beneficial approach. A further advanced BPA strategy augments inoperable patients' clinical status and hemodynamics with CTEPH and a lower mortality rate [11]. The significant adjustments in Mizoguchi et al.'s study show that their advanced BPA procedure initiates the supplement of intravascular ultrasound to decide the ideal balloon size thoroughly and meticulously define the actual size of the target lesions [11]. Evaluating the exact balloon size may limit complications, thus further leading to improved hemodynamic results and reducing the risk of pulmonary reperfusion damage and bursting of the pulmonary artery [11]. Takigami evaluated a specific type of balloon called the Non-Slip Element Percutaneous Transluminal Angioplasty Balloon (NSE PTA). They found no significant pressure gradient enhancements with NSE PTA scoring balloons compared to conventional BPA practices. However, NSE PTA may be suitable for consolidation treatment with sufficient low pulmonary artery pressure after numerous BPA treatments rather than the initial dilation of complete occlusion or severe stenotic lesions [44].

BPA and exercise tolerance: BPA alone improves exercise intolerance, a crucial prognostic forecaster in CTEPH relative to hemodynamic improvements early after the procedure [45]. Fukui et al. evaluated the efficacy of cardiac rehabilitation-initiated BPA in patients with inoperable CTEPH who presented with continuing exercise intolerance and symptoms. The group BPA and succeeding cardiac rehabilitation is a novel treatment approach that improves exercise capacity to near normal levels and heart failure symptoms with a good safety lookout [45]. With continued studies and clinical success. Miura et al. found striking evidence that widespread BPA in inoperable patients after partial hemodynamic improvement is associated with enriched objective exercise, tolerance, and improved QOL scores [46]. Lastly, it was pointed out that complete revascularization by supplementary BPA sessions on lingering lesions, even after PH liberation, could sort out hypoxia or obligations for supplementary pulmonary vasodilators. This data may ameliorate oxygenation and exercise capabilities and reduce the necessity for pulmonary vasodilators or supplemental oxygen therapy [47].

Anticoagulation therapy

Common Concerns in Anticoagulant Therapy

Recurrent venous thromboembolism: Anticoagulant therapy is the standard for long-term CTEPH management with concurrent monitoring for recurrent symptoms [14]. The risk of acute recurrent thrombosis, or “blood clots,” is especially high after cessation of anticoagulant therapy [48]. In CTEPH patients, it is one of the most anticipated concerns during management [49]. Zhu et al.'s literature review identified two studies that have reported recurrent venous thromboembolism (VTE) episodes in patients with CTEPH during their anticoagulation regimen - 1.2/100 patients/year in Japan and 0.76/100 patients/year post-PEA in Britain [49]. The incidence of the Zhu et al.'s cohort was 1.69/100 patients/year. Results from Bunclark et al.'s multicenter study showed recurrent VTE in 20 operable CTEPH patients post-PEA with an overall incidence of 1.17% per person per year [50]. Episodes occurred approximately six months after undergoing PEA [49].

Bleeding: This is another significant concern during the management of operable and inoperable CTEPH patients on anticoagulant therapy. Bunclark et al.’s study explains “major bleeding” as an event in which blood transfusion requires greater than or equal to two red blood cell units at a critical site such as the pericardium, central nervous system, respiratory sites, intraocular and peritoneum [50]. “Nonmajor bleeding” was defined as bleeding that interrupted anticoagulation therapy, required medical intervention, spontaneous physician contact, and generally did not meet the criteria of significant bleeding [50]. According to the review of Zhu et al., bleeding events occurred throughout therapy at an incidence of 0.67% to 5% per person per year [49]. Recent risk factors also include anemia and glucocorticoid use. Anemia was a predisposition to bleeding, and approximately one-third of patients who were concurrently on glucocorticoids displayed gastrointestinal bleeding [49]. A connection has also been made between bleeding events and recurrent thrombosis in that patients who have a high bleeding risk also have an increased risk of thrombosis, which calls for regular monitoring and precaution [51].

Specific anticoagulant drugs

Vitamin-K Antagonists

Lifelong anticoagulation therapy in patients with CTEPH specifically uses VKAs such as warfarin [14]. Warfarin has many FDA-approved clinical uses, such as treating thromboembolic complications from atrial fibrillation, thrombus prophylaxis, and venous thrombosis [52]. Hence, these uses make the drug reliable and is the anticoagulant of choice recommended by physicians. VKA’s general mechanism of action is inhibiting vitamin K epoxide reductase complex I, an enzyme that activates vitamin K in the body and thus prevents it, leading to reduced clotting factor synthesis [52]. The route of administration is oral and is taken both pre- and post-operatively [52].

Direct-Acting Oral Anticoagulants

Examples of commonly used DOACs are clotting factor Xa inhibitors - rivaroxaban, edoxaban, and apixaban [53]. In Zhu et al.’s study, the incidence of recurring venous thromboembolism in patients with CTEPH was 1.84/100 in patients receiving a DOACs regime [49]. Patients who took warfarin reported an incidence of VTE at 1.05/100, whereas patients on DOACS had 1.84/100 [49]. Although the risk of recurrent VTE is slightly higher, these drugs remain in the treatment options. Advantages of using DOACs include less frequent follow-ups, less monitoring, fewer food and drug interactions, and a quick onset and offset times for anticoagulant activity compared to VKAs [53]. A disadvantage, however, is the dependence of hepatic and renal systems for drug clearance [53]. Therefore, these drugs become a contraindication for patients with CTEPH who also have liver or kidney problems.

Heparin

Another substance of choice is heparin sodium intravenous injections, which are anionic mucopolysaccharides called glycosaminoglycans (GAGs) tested for anticoagulant effects [54]. A study by Hirsh et al. showed the mechanism of action of heparin is the inhibition of factor Xa and thrombin [55]. Their study also showed the effects of heparin in humans compared to rabbits. For example, heparin prolongs bleeding time in humans and facilitates blood loss in rabbits [55]. Therefore, separate from its anticoagulant effect, heparin-induced bleeding can also occur. Incidence of recurrent VTE among patients was also found to be 5.4%, and bleeding was fatal in o.2% of patients [55].

Medical Therapy - Riociguat

Riociguat is a soluble guanylate cyclase stimulator, and at the time of the van Thor et al.'s study, it was the only officially registered therapy for CTEPH [13]. It promotes vasodilation by increasing cyclic guanosine monophosphate while improving vascular tone [13]. Short-term results on patient follow-ups after 16 weeks showed improved 6-minute walking distance test, reduced PVR, N-terminal pro-brain natriuretic peptide, and World Health Organization’s functional class [15]. These results were noted across 261 patients who received riociguat therapy in a Ghofrani et al.'s study with inoperable CTEPH or recurrent episodes of PH post-PEA [15]. van Thor et al.’s study was a follow-up analysis on 36 patients with primarily inoperable CTEPH (92%, remaining had recurrent CTEPH) from January 2014 till January 2019 [13]. Survival rates after two, three, and four years were 100%, 94%, and 80%, respectively. Similar to Ghofrani et al.'s study, van Thor et al.'s results also showed improvements in the 6MWD test, World Health Organization functional class, and N-terminal pro-brain natriuretic peptide [13,15].

CTEPH management and COVID-19 relevance

The severe acute respiratory syndrome-coronavirus-2 (SARS-COV2) has become a research topic in many fields, especially cardiovascular and respiratory systems. In the results from Lee et al.'s survey-based analysis, out of 16,979 patients diagnosed with PAH or CTEPH, 50 were also positive for Coronavirus Disease of 2019 (COVID-19) at the beginning of April 2020 [56]. By the fourth week of April, for every 1000 patients with PAH and CTEPH, there was a cumulative incidence of 2.9 COVID-19 cases [56]. These statistics significantly illustrate the significance of relating COVID-19 with CTEPH/PAH as they both compromise the respiratory system. Also, the Akay et al.'s study efficiently demonstrated the challenges healthcare professionals face when dealing with patients suffering from COVID-19, CTEPH, and both COVID-19 and CTEPH [57]. Such challenges include cross-contamination of equipment used therapeutically in both conditions, prioritizing resources such as ventilators and intensive care unit facilities that are eventually needed in both patient populations and risk of viral transmission to patients and professionals [57]. The pulmonary nature of CTEPH already puts this patient population at a high risk and level of vulnerability.

A particular challenge highlighted in Akay et al.'s study was managing the treatment of CTEPH with PEA [57]. As mentioned before, PEA is the most effective therapeutic method, given that patients do not suffer from co-morbidities. However, for PEA to be effective, it needs more extracorporeal membrane oxygenation (ECMO) and ventilators, which have already been in shortage in many countries, including the United States [57]. The virus also worsens the symptoms of CTEPH due to pulmonary restriction, delaying the treatment effects to show [57]. Recurrent thromboembolic episodes may also be triggered due to inflammation and causative hypercoagulation, lack of mobility in the hospital, and prolonged hospital stay because of constant patient monitoring in case viral transmission occurs and management in already co-existing conditions [57].

Limitations

We can account for a handful of limitations in our systematic review. First, our systematic review does not contain a meta-analysis. Secondly, as a systematic review, all analyses solely relied on previous experimental settings. Data from our experimental group with patients may differ, including routine follow-ups from post-operative patients. Lastly, the COVID-19 pandemic has significantly impacted many studies in the past two years - for example, conducting primary research in hospitals was difficult because of severe restrictions. Irrespective of our best work effort, we accept that not all relevant research and studies may have been incorporated in this systematic review, and some articles may be overlooked in the search.

## Conclusions

The benefits of PEA for CTEPH patients are evident, with marked improvements in hemodynamic profile and long-term survival rates. Still, almost half of CTEPH cases are deemed inoperable due to accessibility, complexity, or co-morbidities. Therefore, alternative and effective therapies such as BPA, anti-coagulations, or combined therapy must be considered. BPA can be considered a promising alternative to other procedures, especially in patients deemed inoperable. This hints that better options exist, such as anticoagulant therapy for inoperable patients. 
